# Hypercalcemia due to PTHrp producing pancreas NET: A case report

**DOI:** 10.1016/j.ijscr.2025.111058

**Published:** 2025-02-12

**Authors:** Franziska Brückner, Elisabeth Maurer, Detlef Bartsch, Maxime Schmitt, Anja Rinke

**Affiliations:** a Department of Visceral-, Thoracic- and Vascular Surgery, University Hospital Marburg, Germany; b Department of Pathology, University Hospital Marburg, Germany; c Department of Gastroenterology, University Hospital Marburg, Germany

**Keywords:** Case report, Pancreas NET, PTHrp, Surgery, Review

## Abstract

**Introduction:**

Secretion of parathormone related peptide (PTHrp) is the most common cause of tumor-associated hypercalcemia. This occurs most often in squamous cell carcinomas in the ear nose and throat, bronchial and breast carcinomas. We report a rare case of a PTHrp-producing pancreatic neuroendocrine tumor (pNET) and provide a brief review of the literature.

**Case presentation:**

A 68-year-old female patient with epigastric pain and weight loss was diagnosed with a 11 × 8 × 9 cm tumor in the pancreatic body with infiltration of the splenic vein and consecutive portal vein thrombosis without evidence of distant metastases. An endosonographic fine needle aspiration revealed a neuroendocrine tumor G2 (Ki-67 3 %). Laboratory analyses showed an asymptomatic hypercalcemia and elevated PTHrp. Distal splenopancreatectomy, left adrenalectomy, thrombectomy of the portal vein, cholecystectomy and partial resection of the left renal vein was performed. Histopathologic examination showed a PTHrp-producing NET of the pancreas G2, pT4 pN0 M0 L0 V2 Pn0 R0. Postoperatively serum levels of PTHrp and calcium dropped to normal values.

**Discussion:**

Up to date only 83 cases of PTHrp producing pNETs have been reported in the English literature. Frequently, as in the reported patient, locally advanced or already distantly metastasized tumors were present. In addition to initial drug control of hypercalcemia, surgical treatment in case of R0 resection offers long-term symptom control and should be performed, when possible.

**Conclusion:**

In case of a pancreatic tumor and the simultaneous occurrence of hypercalcemia, the determination of PTHrp should be considered. Surgical resection remains the only curative therapy.

## Highlights

• Rare case of PTHrp producing pancreas NET
• PTHrP-pNETs often indicate advanced disease
• Heterogenous treatment options are described in the currently available literature
• Surgical therapy as the only curative option
• Shows importance of early diagnosis and surgical management in PTHrp producing pNETs with successful outcome even in advanced local disease

## 1 Introduction and importance

Hypercalcemia associated with tumor disease occurs in up to 30 % of all tumor patients [1]. Several pathomechanisms are known, whereby the secretion of the PTH-related peptide (PTHrp) is described to be the most common cause with 80 % of all hypercalcemia in tumor patients [1]. The PTHrp is structurally like conventional PTH as its N-terminal region is closely homologous to PTH and binds to its receptors in the kidney. This leads to increased absorption of calcium and at the same time increased excretion of phosphate resulting in hypercalcemia and hypophosphatemia [1]. The most common tumor entities that produce PTHrp are squamous cell carcinomas of the head, neck, esophagus and lungs, as well as colon carcinomas, but also renal, breast, endometrium and ovarian carcinomas [1]. In very rare cases, PTHrp production was also detected in neuroendocrine neoplasms, whereby the pancreatic neuroendocrine tumors (pNETs) are the most common [2]. Up to date about 83 PTHrp producing pNETs were described in the world literature [2].

Here we present a female patient with hypercalcemia caused by a PTHrp producing pNET and provide a brief review of the literature. The following case has been reported in line with the SCARE criteria [3].

## 2 Case presentation

**Fig. 1:**
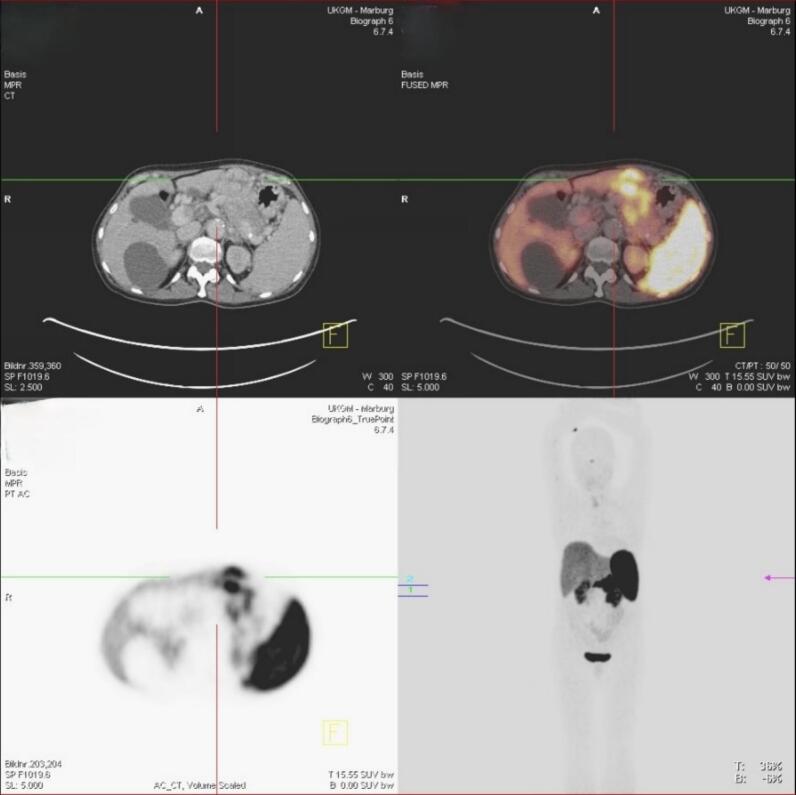
Ga 68-DOTATOC PET-CT: tracer positive tumor in the corpus/tail area of the pancreas with positive lymph nodes.

A 68-year-old female patient presented in our clinic with epigastric pain and a palpable mass in the upper abdomen. The patient complained about epigastric pain as well as an unwanted weight loss of approximately 4–5 kg in the last 6–7 weeks. Laboratory analyses showed an asymptomatic hypercalcemia (3.07 mmol/l) with low intact serum PTH of <6 ng/l (norm value 11–65 ng/l) while serum PTHrp was highly elevated. Further analyses showed an increased serum level for Chromogranin A (202 μg/l, norm value <102 g/l), whereas insulin, gastrin and serotonin serum levels were within normal limits. Ultrasound examination performed by her primary physician documented a suspicious 10 cm sized mass around the pancreas. The triphasic CT-scan showed a 11 × 8 × 9 cm large tumor in the corpus and tail of the pancreas with infiltration of the splenic vein, a tumor thrombus in the portal vein and several lymph node metastases. The endosonographic fine needle biopsy revealed a pancreatic NET G2 (Ki-67 3 %). In the Ga68 DOTATOC-PET-CT the tumor was tracer-positive and there were several metastases-suspected regional lymph nodes, whereas there was no evidence of distant metastases (Fig. 1).

The interdisciplinary tumor conference decided for tumor resection as the primary treatment.

Intraoperatively a large tumor (about 10 × 12 cm) with infiltration of the left adrenal gland, left renal vein and portal vein with extensive bypass circuits and stasis of the spleen was encountered.

**Fig. 2:**
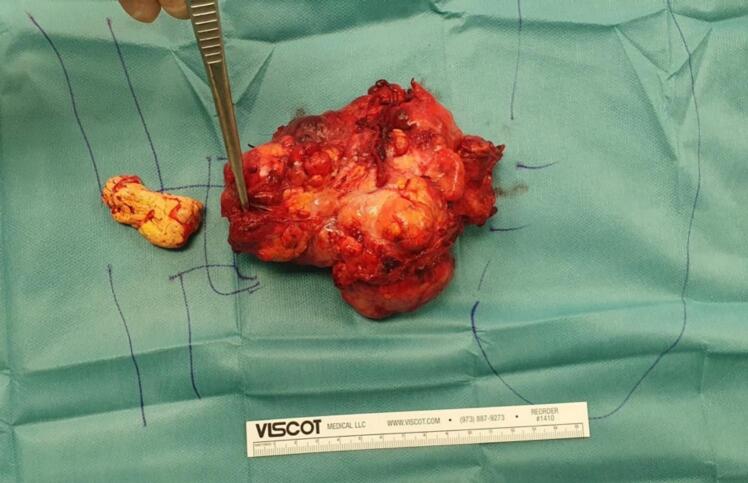
11 × 8 × 9 cm resected tumor in the pancreatic body with infiltration of the splenic vein (en-block splenectomy not shown in this picture).

Open distal splenopancreatectomy with lymphadenectomy, left adrenalectomy, thrombectomy of the portal vein and partial resection of the left renal vein, as well as cholecystectomy due to cholecystolithiasis was performed (Fig. 2). Somatostatin was given perioperatively (pre-, intra- and postoperative).

Intraoperatively 22 packed red blood cells, 18 fresh frozen plasms and 5 platelet concentrates had to be given because of significant bleeding due to fundus and peripancreatic varices.

Overall, the postoperative course was uneventful without complications with only two days postoperatively at the intensive care unit and the patient was discharged on postoperative day 14 with a normalized serum calcium (2.01 mmol/l) and negative serum PTHrp.

**Fig. 3:**
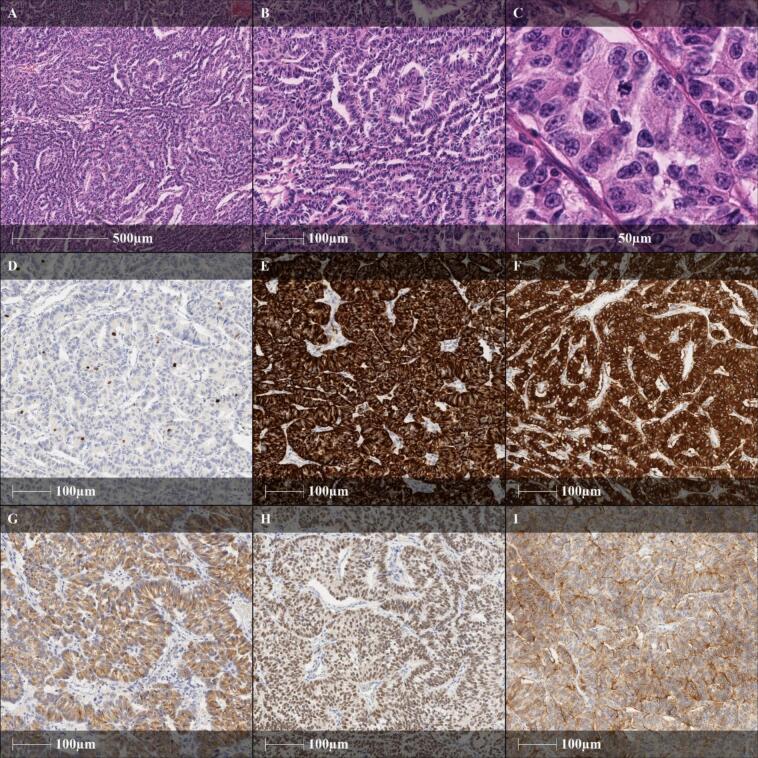
Well differentiated pancreatic neuroendocrine tumor (NET), G2, showing a predominantly garland-shaped growth pattern (A, 10×, H.E.; B, 20×, H.E.) of monotonous cells with small nucleoli demonstrating a „salt and pepper “chromatin and up to 2 mitoses per mm^2^ (C, 100×, H.E.). Ki-67 expression is recognized in 3 % of tumor cells (D, 20×). The neuroendocrine differentiation is immunohistochemically confirmed by a strong membranous cytokeratin-expression (E, 20×, CK MNF 116) in combination with a strong membranous synaptophysin- (F, 20×) and cytoplasmatic chromogranin A-expression (G, 20×). In further immunohistochemical examination the tumor cells show nuclear expression of islet 1 (H, 20×), membranous expression of SSTR2A (I, 20×). NET: neuroendocrine tumor; CK MNF116: cytokeratin, clone: MNF116; SSTR2A: somatostatin-receptor 2A.

The histology confirmed a PTHrp-producing NET of the pancreas G2 (Ki-67 3 %), pT4 pN0 M0 L0 V2 Pn0 R0 (Fig. 3).

Two months after surgery the patient showed no symptoms with normal serum calcium (2.33 mmol/l) and negative PTHrp.

The Ga-68-DOTATOC-PET/CT 4 and 12 months after the surgery revealed no evidence of local relapse or metastasis. In the last follow-up 12 months after surgery, the patient was still asymptomatic with normal serum calcium (2.48 mmol/l) while PTH was with 87 ng/l (norm 11–65 ng/l) slightly increased. Although no morphological correlate for recurrence was encountered, we started the treatment with somatostatin analogues (SSA).

## 3 Clinical discussion

Humoral hypercalcemia of malignancy (HHM) is often caused by paraneoplastic expression of PTHrp (80 % of hypercalcemia in cancer patients) [1]. The first case of PTHrp-producing pancreas NET was described in 1961 [2]. Since then, about 83 cases are documented in the world literature [2], of whom we review a series of 33 cases since 1990 (see Table 1, Table 2).

**Table 1:** Characteristics of published patients with PTHrp-pNET.

Patient Nr. (reference)	Gender	Age at DX	PTHrp elevated at DX	Serum calcium at DX (mmol/l)	SRS imaging (pos/neg)	ENETS stage at DX	Grading	Ki-67 (%)
1 [10]	m	59	Yes	4.71	pos	III	n.d.	n.d.
2 [11]	f	62	Yes	3.14	pos	IV	G3	30
3 [12]	m	34	Yes	3.8	pos	III	n.d.	n.d.
4 [8]	f	49	Yes	3.4	n.d.	IV	G1	<2
5 [8]	m	53	Yes	n.d.	n.d.	IV	n.d.	n.d.
6 [8]	m	52	Yes	3.7	n.d.	IV	G2	<5
7 [8]	m	54	Yes	4.35	n.d.	IV	G1	2
8 [13]	f	25	Yes	5.52	n.d.	IV	G2	5
9 [14]	m	30	Yes	n.d.	n.d.	IV	n.d.	n.d.
10 [14]	f	60	Yes	n.d.	n.d.	IV	n.d.	n.d.
11 [15]	f	25	Yes	5.9	pos	n.d.	n.d.	n.d.
12 [16]	f	68	Yes	2.9	pos	III	G2	8 %
13 [17]	f	58	Yes	3.65	n.d.	IV	G1/G2	n.d.
14 [18]	f	54	Yes	>4	pos	IV	G1	<1
15 [19]	m	48	Yes	3.0	pos	IV	n.d.	n.d.
16 [20]	m	58	Yes	2.95	pos	IV	G2	4
17 [5]	m	41	n.d.	2.99	n.d.	IV	n.d.	n.d.
18 [5]	m	58	n.d.	3.64	n.d.	IV	G1	n.d.
19 [5]	f	40	n.d.	3.33	n.d.	IV	n.n.	n.d.
20 [5]	m	52	n.d.	3.35	n.d.	IV	G3	n.d.
21 [5]	m	61	n.d.	2.89	pos	IV	G1/G2	n.d.
22 [5]	m	38	n.d.	2.67	n.d.	IV	G1	n.d.
23 [5]	f	42	n.d.	3.53	n.d.	IV	n.d.	n.d.
24 [5]	f	51	n.d.	2.93	n.d.	IV	n.d.	n.d.
25 [5]	f	49	n.d.	3.57	n.d.	IV	G1	n.d.
26 [9]	m	64	Yes	2.7	n.d.	IV	G1	2,5
27 [21]	f	25	Yes	3.38	pos	n.d.	n.d.	n.d.
28 [21]	f	44	Yes	2.92	pos	IV	n.d.	n.d.
29 [21]	m	26	Yes	2.87	pos	IV	n.d.	n.d.
30 [21]	f	64	Yes	2.97	pos	IV	n.d.	n.d.
31 [21]	f	34	Yes	3.38	pos	IV	n.d.	n.d.
32 [22]	m	51	Yes	3.74	pos	IV	G1	<3
33 [23]	f	53	Yes	2.84	pos	IV	G1	<1
34 (our patient)	f	68	Yes	3.07	pos	III	G2	3

n.d.: no data.

**Table 2:** Performed treatment sequences in published PTHrp pNETs.

Patient nr. (references)	Resection of primary and metastases	R0/R1/R2 resection	SSA	PRRT	Chemotherapy	INF-alpha	TACE	Follow-up (months)	Disease status
1 [10]	3rd	R1	1st			2nd		49	AWD
2 [11]	2nd	R2	1st		1st			6	AWD
3 [12]	No surgery				1st			28	DOD
4 [8]	No surgery		1st	2nd				36	AWD
5 [8]	No surgery				2nd		1st	96	AWD
6 [8]	1st	R0					2	60	AWD
7 [8]	No surgery		2nd	1st	3rd			15	AWD
8 [13]	1st	n.d.	2nd		3rd		2nd	180	AWD
9 [14]	1st	R0						2	AWD
10 [14]	1st	R2				2nd		12	DOD
11 [15]	1st	R0						24	NED
12 [16]	1st	R0						n.d.	NED
13 [17]	1st	R0						19	NED
14 [18]	No surgery		1st					3	AWD
15 [19]	No surgery		1st	2nd	1st			50	AWD
16 [20]	No surgery		1st	3rd	1st	2nd	4th	120	AWD
17 [5]	No surgery							n.d.	
18 [5]	No surgery							n.d.	
19 [5]	No surgery							n.d.	
20 [5]	No surgery							n.d.	
21 [5]	No surgery		1st	1st	2nd			100	AWD
22 [5]	No surgery							n.d.	
23 [5]	No surgery							n.d.	
24 [5]	No surgery							n.d.	
25 [5]	1st	R0	3rd	2nd			4th	198	DOD
26 [9]	2nd	n.d.	3rd	5th	1st		4th	54	DOD
27 [21]	1st	R0	2nd					72	AWD
28 [21]	No surgery		1st					n.d.	
29 [21]	No surgery		1st					n.d.	
30 [21]	No surgery		1st					n.d.	
31 [21]	1st	R2	2nd					n.d.	
32 [22]	No surgery		1st		1st			6	AWD
33 [23]	No surgery		1st					n.d.	
34 (our patient)	1st	R0	2nd					12	NED
Total *n* = 34	14		12	6	10	3	5		

1st: first therapy, 2nd: second therapy, 3rd: third therapy etc.

AWD: alive with disease, DOD: dead of disease, NED: no evidence of disease.

The clinical characteristics, treatment regimens and outcome of these patients were compared. Overall, the PTHrp-producing pNETs showed no gender specific predominance (17 women, 16 men) and the average age of onset was 49 years (Table 1). Two of these 33 cases were diagnosed with a MEN-1 syndrome.

As in our patient, all PTHrp-producing pNETs were well-differentiated tumors mostly G1 or G2 with a Ki-67 index of 1 to 30 % (Table 1).

As HHM is known to be a sign of far advanced solid unresectable distant metastases [4] this also seems to be the case in pNETs. Thirty of 34 (88 %) patients with PTHrp-producing pNETs had an advanced tumor stage IV with distant metastases at the time of diagnosis, whereas only 4 patients, including the presented, had stage III tumors, of whom only 2 underwent primary surgery in curative intention (see Table 2).

Five years survival of stage IV PTHrp-producing pNETs with available follow-up data is about 70 %, and thus comparable to stage IV NF-pNETs and insulinoma with reported five years survival of 83 % [5] (see also Table 2). Patients with PTHrp-producing pNETs seem to have a longer median survival of about 23,5 months [2] compared to other PTHrp-producing solid tumors with a median survival time of 6 weeks [4].For the reported PTHrp-producing pNET who underwent surgery (*n* = 14), the biochemical cure rate after 2 years was 28 % (4 of 14 patients, Table 2). Our patient remained without imageable evidence of disease 12 months after surgery.

In pNETs with locally advanced tumor stage, a neoadjuvant chemotherapy could be performed to reduce the tumor burden and to allow consecutive surgery [6]. This is also a possible treatment option for locally irresectable or borderline resectable PTHrp producing pNETs, as it was performed in 2 cases (Table 2). In the presented case the multidisciplinary tumor board voted for primary resection, which could achieve a R0 resection.

One of the reasons for the prolonged survival time could be the usually slow tumor growth, especially in well-differentiated pNETs [7], as well as the nowadays available multiple treatment options in surgically non-curable tumors, such as somatostatin analogues (SSA), different chemotherapy regimens such as 5-FU and streptozotocin or temozolomide and capecitabine and/or peptide receptor radionuclide therapy (PRRT).

Somatostatin analogues bind to somatostatin receptors on the tumor itself which reduces endocrine symptoms but also retards tumor growth [8]. In metastasized PTHrp-producing pNET SSA-therapy leads to decreasing PTHrp serum levels and therefore decreasing calcium levels [9]. SSA-therapy should be used as first-line-treatment in patients with a non-resectable G1/G2 tumor with a Ki-67 < 10 % [6]. In case of R1-situation or metastases after surgery it can be used as palliative treatment for symptom control.

For non-resectable pNETs G2 and Ki-67 > 10 % the first line-treatment is chemotherapy with streptozotocin and 5-Fluorouracil or temozolomide and capecitabine [6]. In case of tumor progression sunitinib or everolimus could be also used as antiproliferative treatment [6].

In case of liver metastases cytoreduction could be performed by peptide receptor radionuclide therapy (PRRT), where intravenous SSA is combined with a radioactive particle (e.g., 177-Lutetium-DOTATOC). Also, ablative cytoreductive methods like radiofrequency ablation (RFA) or transarterial chemo-/embolization (TAE/TACE) could be used in case of non-resectable liver metastases.

All above mentioned treatment options in the published cases were used in different sequences and treatment was thus quite heterogenous (see Table 2). Therefore, patients with these extremely rare PTHrp-pNET should be treated in expert centers based on the decision of a multidisciplinary tumor board.

## 4 Conclusion

Hypercalcemia is a very rare symptom in patients with pancreatic NETs. In case of hypercalcemia and normal serum levels of intact PTH in patients with a pNET, the serum level of PTHrp should be assessed as well. PTHrp-pNET should be completely resected whenever possible, since it leads to long-term symptom control and survival.

## Patient consent

Written informed consent was obtained from the patient for publication and any accompanying images. A copy of the written consent is available for review by the Editor-in-Chief of this journal on request.

## Ethical approval

After consultation with the ethic committee an ethical approval was not required since this paper only describes a medical case after guideline-orientated therapy.

## Guarantor

Prof. Dr. Med. D. Bartsch

## Funding

N/A

## Author contribution

Franziska Brückner: 1st author, data collection, data analysis and interpretation, writing the paper

Elisabeth Maurer: contributor

Detlef Bartsch: contributor, (1)writing the paper, data analysis

Maxime Schmidt: data analysis, contributor

Anja Rinke: comntributor

## Conflict of interest statement

N/A
